# *DICER1* RNase IIIb domain mutations trigger widespread miRNA dysregulation and MAPK activation in pediatric thyroid cancer

**DOI:** 10.3389/fendo.2023.1083382

**Published:** 2023-02-21

**Authors:** Julio C. Ricarte-Filho, Victoria Casado-Medrano, Erin Reichenberger, Zachary Spangler, Michele Scheerer, Amber Isaza, Julia Baran, Tasleema Patel, Suzanne P. MacFarland, Garrett M. Brodeur, Douglas R. Stewart, Zubair Baloch, Andrew J. Bauer, Jonathan D. Wasserman, Aime T. Franco

**Affiliations:** ^1^ Division of Endocrinology and Diabetes, Children’s Hospital of Philadelphia, University of Pennsylvania, Philadelphia, PA, United States; ^2^ Department of Biomedical and Health Informatics, Children’s Hospital of Philadelphia, Philadelphia, PA, United States; ^3^ Division of Oncology, Children’s Hospital of Philadelphia, University of Pennsylvania, Philadelphia, PA, United States; ^4^ Cancer Predisposition Program, Children’s Hospital of Philadelphia, University of Pennsylvania, Philadelphia, PA, United States; ^5^ Abramson Cancer Center, University of Pennsylvania, Philadelphia, PA, United States; ^6^ Division of Cancer Epidemiology and Genetics, National Cancer Institute, Rockville, MD, United States; ^7^ Department of Pathology and Laboratory Medicine, Children’s Hospital of Philadelphia, University of Pennsylvania, Philadelphia, PA, United States; ^8^ Division of Endocrinology, The Hospital for Sick Children, Toronto, ON, Canada

**Keywords:** *DICER1*, mutation, RNAse IIIb, pediatric, thyroid, cancer, microRNA, MAPK

## Abstract

DICER1 is a highly conserved RNase III endoribonuclease essential for the biogenesis of single-stranded mature microRNAs (miRNAs) from stem-loop precursor miRNAs. Somatic mutations in the RNase IIIb domain of DICER1 impair its ability to generate mature 5p miRNAs and are believed to drive tumorigenesis in DICER1 syndrome-associated and sporadic thyroid tumors. However, the *DICER1*-driven specific changes in miRNAs and resulting changes in gene expression are poorly understood in thyroid tissue. In this study, we profiled the miRNA (n=2,083) and mRNA (n=2,559) transcriptomes of 20 non-neoplastic, 8 adenomatous and 60 pediatric thyroid cancers (13 follicular thyroid cancers [FTC] and 47 papillary thyroid cancers [PTC]) of which 8 had *DICER1* RNase IIIb mutations. All *DICER1-*mutant differentiated thyroid cancers (DTC) were follicular patterned (six follicular variant PTC and two FTC), none had lymph node metastasis. We demonstrate that *DICER1* pathogenic somatic mutations were associated with a global reduction of 5p-derived miRNAs, including those particularly abundant in the non-neoplastic thyroid tissue such as let-7 and mir-30 families, known for their tumor suppressor function. There was also an unexpected increase of 3p miRNAs, possibly associated with *DICER1* mRNA expression increase in tumors harboring RNase IIIb mutations. These abnormally expressed 3p miRNAs, which are otherwise low or absent in *DICER1*-wt DTC and non-neoplastic thyroid tissues, make up exceptional markers for malignant thyroid tumors harboring *DICER1* RNase IIIb mutations. The extensive disarray in the miRNA transcriptome results in gene expression changes, which were indicative of positive regulation of cell-cycle. Moreover, differentially expressed genes point to increased MAPK signaling output and loss of thyroid differentiation comparable to the RAS-like subgroup of PTC (as coined by The Cancer Genome Atlas), which is reflective of the more indolent clinical behavior of these tumors.

## Introduction

DICER1 is a highly conserved RNase III endoribonuclease essential for the biogenesis of single-stranded mature microRNAs (miRNAs) from stem-loop precursor miRNAs (pre-miRNAs) (1). It possesses two tandem endonuclease domains, RNase IIIa and RNase IIIb, which selectively cleaves 3p miRNA and 5p miRNA from the 3′ and 5′ pre-miRNA, respectively (2, 3). Therefore, each pre-miRNA will generate two mature miRNA strands (5p and 3p) that contain different mRNA-targeting sequences. The produced mature miRNAs are loaded into the RNA-induced silencing complex (RISC) and suppress gene expression by binding to the 3′ untranslated region (3′-UTR) of the target mRNAs (4). By targeting more than half of protein-coding genes, miRNAs are involved in virtually all developmental and pathological processes in animals, including malignant neoplasms (5, 6).

Pathogenic and likely pathogenic germline *DICER1* variants are associated with DICER1-related tumor predisposition, a syndrome characterized by an increased risk for pleuropulmonary blastoma (PPB), pulmonary cysts, thyroid gland neoplasia (multinodular goiter, adenomas, and/or thyroid cancer), ovarian tumors (Sertoli-Leydig cell tumor, gynandroblastoma, and sarcoma), cystic nephroma, and Wilms tumor (7). DICER1-associated tumors typically harbor a germline loss-of-function (LOF) variant (nonsense, frame-shift, rarely missense; copy number changes are rarely observed) and a tumor-specific missense mutation in the DICER1 RNase IIIb domain affecting one of the five hotspot codons that encode key amino acids in the metal biding catalytic cleft of the nuclease domain: E1705, D1709, G1809, D1810, and E1813 (8–11). DICER1-related tumor predisposition therefore represents an unusual form of Knudson’s two-hit hypothesis, since the somatic mutation affects the ability of DICER1 RNase IIIb domain to process 5p miRNAs, while keeping its capacity to generate 3p miRNAs by the preserved RNase IIIa domain (2, 3, 12). This change in 5p:3p strand ratio alters the miRNA repertoire of tumor cells and is predicted to drastically alter gene expression.

Disruption of *Dicer1* in mice leads to lethality early in embryonic development (13). Thyroid-specific inactivation of *Dicer1* generates mice with severe hypothyroidism and marked loss of differentiation markers (*Tg*, *Nis*, *Tshr* and *Tpo*) (14), further emphasizing its role in thyroid pathogenesis. Indeed, *DICER1* hotspot mutations have been reported in human thyroid neoplasms of follicular cell origin, including adenomas, papillary thyroid carcinoma (PTC), follicular thyroid carcinoma (FTC), and poorly differentiated thyroid cancers (PDTC) (15–22). The effects of *DICER1* somatic mutations on miRNA biogenesis and the associated gene expression changes that are relevant for thyroid tumorigenesis are poorly understood. Experimental data suggest that the pathogenicity of somatic mutations in *DICER1* is dependent on the cellular context and controlled by tissue-specific miRNAs and mRNAs (23, 24). Therefore, genome-wide miRNA/mRNA expression studies will be fundamental to determine tissue-specific miRNA signatures associated with *DICER1*-driven thyroid tumors and identify oncogenic pathways activated by RNase IIIb hotspot mutations. In this study, we aimed to evaluate the effects of *DICER1* RNase IIIb mutations on miRNA and mRNA transcriptomes of differentiated thyroid cancers. We propose molecular mechanisms (gene networks and signaling pathways) underlying the tumorigenesis of follicular thyroid cells harboring *DICER1* somatic mutations. We also uncover potential miRNA biomarkers for *DICER1*-driven malignant tumors that may be valuable in the clinical setting.

## Material and methods

### Patient samples

Tumor samples were obtained from the Children’s Hospital of Philadelphia (CHOP) and Hospital for Sick Children, Toronto, Ontario, Canada. This study included archived Formalin-Fixed Paraffin-Embedded (FFPE) samples from 20 non-neoplastic thyroids, 8 adenomatous nodules and 60 sporadic well-differentiated follicular derived thyroid cancers (DTC): 47 PTCs and 13 FTCs. Demographic information including age and sex was collected from each patient in addition to histopathologic results and tumor staging. Ethics approvals for collection and use of the patient samples were obtained from the CHOP Institutional Review Board as part of the Child and Adolescent Thyroid Consortium (CATC) Biorepository study (IRB# 20-018240).

### miRNA and mRNA targeted next-generation sequencing (NGS) analysis

Tissue was cut into 5µm serial sections and mounted on glass microscope slides. Diagnosis was confirmed by a board-certified pathologist specialized in thyroid disease and regions of interest representing tumor foci on an H&E slide were selected and circled. Samples were sent to HTG Molecular Diagnostics in Tucson, AZ, and regions of interest were microdissected under a Leica LMD6500 laser capture microscope (LCM). Each sample was immediately suspended in EdgeSeq lysis buffer (HTG Molecular Diagnostics, Tucson, AZ) and miRNA/mRNA profiling was carried out using one FFPE tissue slide from each sample for each assay as previously described (25). miRNA and mRNA expression were profiled using HTG Edgeseq miRNA whole transcriptome assay and HTG EdgeSeq Oncology Biomarker Panel (OBP), respectively. HTG EdgeSeq system is a NGS application that measures gene expression without the need for extracting RNA. HTG Edgeseq miRNA whole transcriptome assay measures the expression of 2,083 human miRNAs described in the miRBase v20 database. HTG EdgeSeq OBP measures the expression of 2,559 genes associated with tumor biology, including 15 housekeeping genes. All miRNAs and mRNAs screened in this study are listed in **Supplemental Tables 1**, **2**, respectively. Briefly, the workflow entailed automated, extraction-free sample preparation, quantitative nuclease protection using the EdgeSeq processor, and library generation and sequencing. The HTG EdgeSeq Parser (HTG Molecular, Tucson, AZ, USA) was used to align the FASTQ files to the probe list to collate the data. Data were provided as data tables of raw, quality control (QC) raw, counts per million (CPM), and median normalized counts.

### Bioinformatic analysis

The DESeq2 data normalization, analyses, and statistical comparisons between benign thyroid tissue and *DICER1*-mutant thyroid tumors were performed using the HTG EdgeSeq Reveal software version: 4.0.1. DESeq2 normalized data were logarithmically scaled for data visualization. Plots were created using GraphPad prism 9 (GraphPad software Inc., La Jolla, CA). TCGA RNA-Seq expression data for adult PTCs and non-neoplastic thyroid tissues were obtained from http://tcga-data.nci.nih.gov and http://gdac.broadinstitute.org/in September 2022. Functional enrichment analysis was performed using g:Profiler to search for genes significantly over-represented in the list of DEG, as compared to all background genes included in the HTG OBP panel (26). Predicted targets for different miRNAs were obtained from TargetScan Human release 8.0 (https://www.targetscan.org/vert_80/). Only predicted targets with a total context score <−0.2 were included in the analysis.

### Biostatistical analysis

Differential expression was calculated using DESeq2 and only differentially expressed mRNAs and miRNAs with a false-discovery rate (FDR) < 0.05 were included. T-test analysis and One-Way ANOVA were performed with GraphPad prism 9. A two-sided *p* value < 0.05 was considered statistically significant.

## Results

### Pediatric thyroid tumors with *DICER1* RNase IIIb mutations are associated with less invasive phenotype

In thyroid, *DICER1*-driven PTCs are infrequent in adult (0-0.4%) but comparatively more prevalent in pediatric PTCs and FTCs (5-10%) (15, 17, 20, 27, 28). Despite its overall low prevalence, we identified 12 cases of DTC with *DICER1* hotspot mutations as part of the Child and Adolescent Thyroid Consortium (CATC) multi-institutional collaborative research program (nine from CHOP and three from Sick Kids). Detailed histopathologic features and molecular alterations of these 12 cases are summarized in **Table 1**. Four of these 12 have been previously reported and published: cases ID# 6, 7, 11, and 12 (20, 29). The 12 cancer cases with mutations in the RNase IIIb domain of *DICER1* included nine females (75%) with mean age of 13.7 years (*SD =* 2.0) at surgery. DTC harboring *DICER1* mutations were all of the follicular type: four FTCs and six follicular variant PTCs (fvPTC). None had extrathyroidal extension, lymph node or distant metastasis. Loss of function defects were found in six cases (three loss of heterozygosity (LOH) and three inactivating variants). To determine whether *DICER1* mutations were somatic or germline, DNA from non-neoplastic thyroid tissue from the same cases was analyzed by conventional Sanger sequencing and somatic alterations are highlighted in bold in **Table 1**. All the RNase IIIb mutations tested and the three inactivating variants reported here (S125*, L777fs, G661Vfs*24) were found to be somatic by Sanger sequencing (**Supplemental Figure 1**). In 3 cases, *DICER1* RNase IIIb alteration was revealed due to the miRNA signature (further discussed in the next section), but no tissue was available to confirm the mutational status. In one case (case 9), *DICER1* positivity was detected by a commercially available somatic thyroid oncogene panel but the specific codon was not reported.

**Table 1:** Demographic and histopathological features of pediatric thyroid cancers with *DICER1* RNase IIIb domain alterations.

ID#	Sex	Age	Histology	TNM	Variant	
					LOF	RNase IIIb
1	M	10.8	fvPTC	T3N0M0	NA	miRNA sig.
2	M	11.4	fvPTC	T1bN0M0	NA	miRNA sig.
3	F	15.7	fvPTC	T2N0M0	NA	p.D1709N
4	M	10.4	fvPTC	T3N0M0	p.S125*	**p.E1813K**
5	F	14.2	FTC	T2N0Mx	NA	p.D1709V
6	F	14.6	fvPTC	T2N0Mx	**p.L777fs**	**p.E1705K**
7	F	15.1	FTC	T2N0M0	p. G661Vfs*24	**p. E1813K**
8	F	14.7	fvPTC	T1aN0aMx	LOH	**p.E1813K**
9	F	14.6	FTC	T1aN0Mx	NA	DICER1+
10	F	16.6	FTC	NA	NA	miRNA sig.
11	F	14.7	fvPTC	T2N0aM0	LOH	**D1810Y**
12	F	11.8	fvPTC	T2N0bM0	LOH	**E1813D**

fvPTC, follicular-variant PTC; FTC, Follicular Thyroid Carcinoma; LOF, Loss-of-function; LOH, Loss of Heterozygozity; miRNA sig., miRNA signature; NA, not available. Mutations depicted in bold have been confirmed to be somatic by DNA sequencing. DICER1 RNase IIIb mutations of cases 6, 7, 11, and 12 have been previously reported.

### DTC with *DICER1* RNase IIIb mutations is associated with widespread miRNA dysregulation

**Figure 1:**
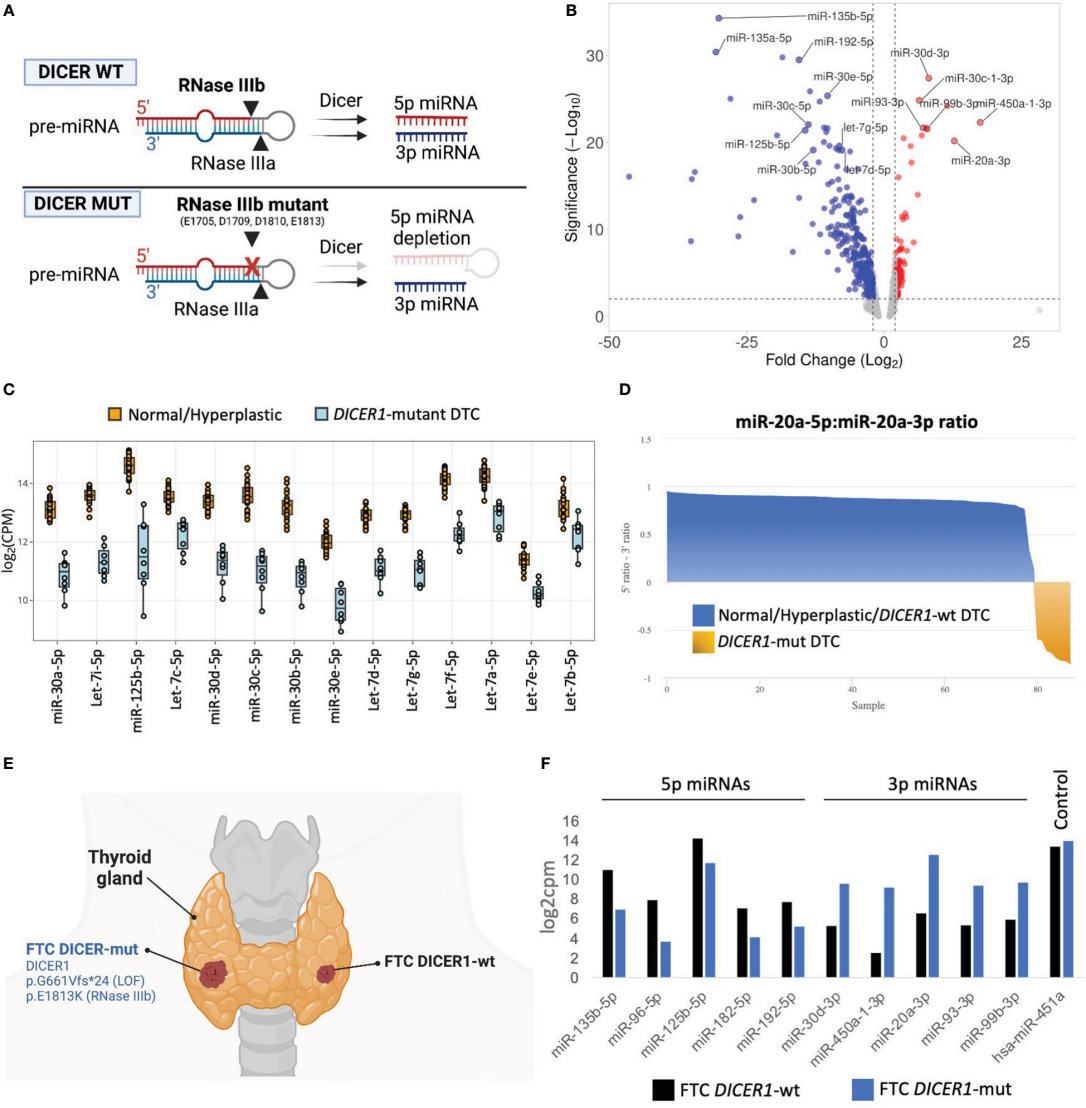
miRNA dysregulation in thyroid cancers harboring hotspot mutations in *DICER1* RNase IIIb domain. **(A)** Scheme illustrating the mechanism by which the two RNase III domains of DICER1 cleave a pre-miRNA hairpin, and for specific loss of 5p miRNAs in RNase IIIb hotspot mutants. **(B)** Volcano plot highlighting miRNAs differentially expressed between 8 differentiated thyroid cancers harboring RNase IIIb mutations and 26 non-neoplastic/hyperplastic cases. Analysis shows general downregulation of 5p miRNAs and upregulation of 3p miRNAs. **(C)** Differential expression of selected miRNAs well-known for their tumor suppressor function in cancer, including let-7 and miR-30 families and miR-125b-5p. **(D)** Analysis of miR-20a pre-miRNA strand processing (5p:3p ratio) across normal/hyperplastic, *DICER1*-wt tumors and *DICER1*-mut tumors. The tumors with hotspot mutations of *DICER1* (shown in yellow) exhibit selective defects in processing 5p miRNA strands (but not 3p), leading to overall decrease in 5p:3p strand ratios. **(E)** Scheme of patient diagnosed with bilateral follicular thyroid cancer (FTC), one with *DICER1* RNase IIIb and one, *DICER1*-wt. Created with BioRender.com. **(F)** The 2 FTCs from the same patient show remarkable differences in miRNAs levels. *DICER1*-mut FTC (right lobe) shows reduction in 5p miRNAs and upregulation of 3p miRNAs when compared to *DICER1*-wt FTC (left lobe).

FFPE tissue specimens were available for eight of 12 cases with DTCs harboring *DICER1* RNase IIIb domain mutations. To determine the impact of DICER1 hotspot mutations on miRNA synthesis, we analyzed the differential miRNA expression in *DICER1*-mut DTC (six fvPTC and two FTC) vs non-neoplastic/adenomatous thyroids (n=26) using the HTG Edgeseq miRNA whole transcriptome assay which evaluates the expression of 2,083 human mature miRNAs. The *DICER1* hotspot mutations in the RNase IIIb domain (E1705, D1709, D1810, and E1813) have been shown to impair the ability of *DICER1* to generate mature 5p miRNAs as illustrated in **Figure 1A** (8). On par with its role in miRNA biogenesis, we show that tumors harboring these mutations demonstrate clear reduction of 5p miRNAs (regardless of the specific mutation site of the RNase IIIb domain), including those abundantly expressed in the non-neoplastic thyroid tissue and associated with tumor suppressor function in thyroid cancer such as let-7 and mir-30 families and mir-125b (30–32)(**Figures 1B, C**). Previous mechanistic studies have shown that RNase IIIa and IIIb activities are distinct and can be uncoupled (2, 33). We found that levels of 3p miRNAs were markedly increased when compared to non-neoplastic thyroid tissue and benign hyperplastic lesions (**Figure 1B**). This striking decrease in 5p:3p strand ratios caused by the RNase IIIb hotspot mutations is further illustrated by the differential processing of miR-20a pre-miRNA in *DICER1*-mut tumors (**Figure 1D**). While benign thyroids and *DICER1*-wt DTC show predominance of miR-20a-5p, *DICER1*-mut DTC have a prominent reduction in 5p:3p strand ratios and express mostly the 3p strand (miR-20a-3p). Moreover, we report a patient with bilateral FTC, with the carcinoma on the right lobe harboring biallelic *DICER1* alterations (p.G661Vfs*24, p.E1813K) while the carcinoma on the left lobe was *DICER1*-wt (**Figure 1E**). Predictably, the *DICER1*-mutant FTC showed reduced levels of 5p miRNAs and increased levels of 3p miRNAs compared to the *DICER1*-wt FTC (**Figure 1F**). miR-451a is the only known miRNA for which maturation has been shown to be DICER1-independent (34). Indeed, expression levels of miR-451a were similar between the two tumors, on par with the non-requirement for DICER1 for its processing, and emphasizing the causal effect of the *DICER1* on the downstream miRNA changes.

### *DICER1*-mutant DTC exhibit unique miRNA signature

**Figure 2:**
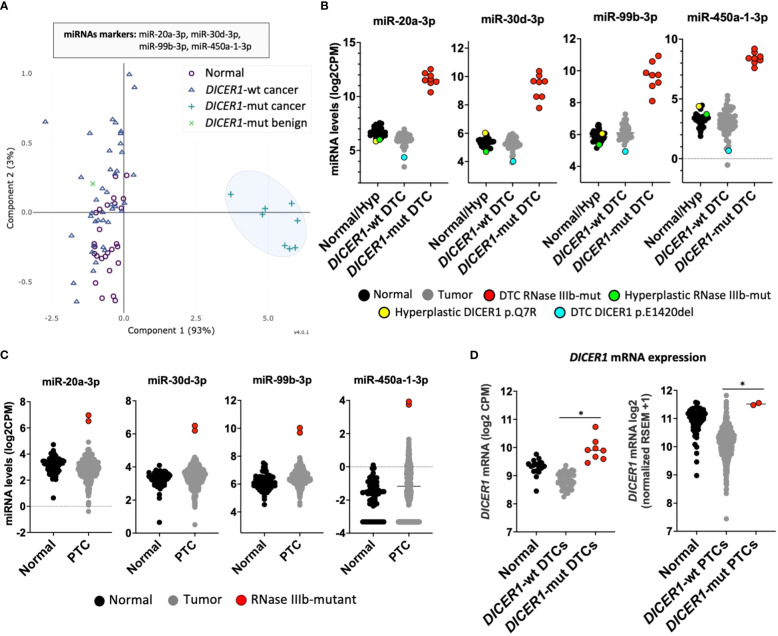
*DICER1*-mutant thyroid cancers exhibit unique miRNA signature. **(A)** Principal component analysis (PCA) of miRNA expression profiles from differentiated thyroid cancer (DTC) and non-neoplastic/hyperplastic shows that a set of four miRNAs (mir-20a-3p, mir-30d-3p, mir-99b-3p, and miR-450a-1-3p) can identify DTC with RNase IIIb mutation. **(B)** Expression of 3p miRNA markers: mir-20a-3p, mir-30d-3p, mir-99b-3p, and miR-450a-1-3p in 28 non-neoplastic/hyperplastic thyroids, 52 *DICER1*-wt DTC and 8 *DICER1*-mutant DTC. **(C)** Expression of miRNA markers: mir-20a-3p, mir-30d-3p, mir-99b-3p, and miR-450a-1-3p across 59 non-neoplastic thyroids and 506 papillary thyroid cancers (PTC) from the Thyroid Cancer The Cancer Genome Atlas (TCGA). Only the 2 PTC cases in the TCGA with *DICER1* RNase IIIb domain mutations show overexpression of the 3p miRNA markers. **(D)** Expression of *DICER1* mRNA in non-neoplastic/hyperplastic thyroid, *DICER1*-wt tumors and *DICER1*-mut tumors from our cohort (left) and Thyroid Cancer TCGA cohort (right). Asterisk represent p < 0.05.

We next sought to determine whether this unique miRNA signature of tumors with RNase IIIb mutations could be used to identify thyroid cancers harboring *DICER1* hotspot mutations. Principal component analysis (PCA) revealed that a classifier with as few as four 3p-miRNA markers (miR-20a-3p, miR-30d-3p, miR-99b-3p, and miR-450a-1-3p) could efficiently distinguish *DICER1*-mut DTC (regardless of histology: PTC or FTC) from non-neoplastic/benign hyperplastic and *DICER1*-wt PTCs/FTCs (**Figure 2A**). Two cases of benign hyperplastic nodules (adenomatous follicular hyperplasia) harboring *DICER1* variants, one case with biallelic *DICER1* alterations (p.E1813G, p.T847delinsFHKHS) and one with a variant of unknown significance (p.Q7R) showed an interesting pattern of miRNA expression. Both cases showed decreased expression of 5p miRNAs, comparable to that observed in *DICER1*-mut DTCs (**Supplemental Figure 2**). However, these *DICER1*-mut benign nodules had no increase of 3p miRNAs as observed for *DICER1*-mut DTCs (**Figure 2B**), indicating these 3p miRNAs are specifically upregulated in malignant cases with RNase IIIb mutation. One case of DTC harboring the *DICER1* p.E1420del (VUS) mutation had no significant differences in miRNA levels. Therefore, 3p miRNAs are not only valuable to classify RNase IIIb mutants as benign or malignant but could also be causally involved in *DICER1*-driven malignant progression. The validity of these four miRNAs biomarkers was subsequently tested using the TCGA cohort comprising 496 adult PTC patients for which we know there are two cases with *DICER1* hotspot mutations: p.E1813G and p.D1810H (17). The expression of these four miRNAs is also dramatically increased in two adult PTCs harboring *DICER1* RNase IIIb mutation when compared to 59 non-neoplastic and 496 *DICER1*-wt PTCs (**Figure 2C**). One PTC case driven by NRAS p.Q61R and with a co-occurring DICER1 p.R1906S mutation (outside of the RNase IIIb domain) had no increase in the expression of these markers. We next sought to determine whether *DICER1* mRNA expression was altered in any of the tumors. In both cohorts (CHOP and TCGA), we found *DICER1* mRNA expression to be downregulated in *DICER1*-wt DTC compared to non-neoplastic cases. On the other hand, DTC with *DICER1* RNase IIIb mutations were associated with increased expression of *DICER1* mRNA and could explain, at least in part, the increased levels of 3p miRNAs observed in these cases (**Figure 2D**). The differential expression of selected 5p and 3p miRNAs (let-7i-5p, miR-30a-5p, miR-20a-3p and miR-99b-3p) was further validated by quantitative PCR in four *DICER1*-mut DTC and matched normals – cases ID# 5, 6, 7 and 8 from **Table 1** (**Supplemental Figure 3**).

### *DICER1* hotspot mutations are associated with increased MAPK output and loss of thyroid differentiation

**Figure 3:**
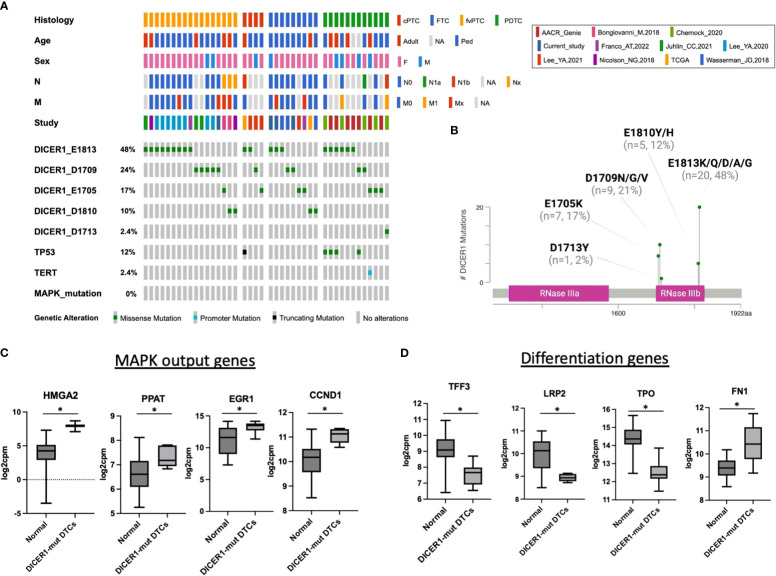
*DICER1* RNase IIIb mutations are associated with increased MAPK output and loss of differentiation. **(A)** Oncoprint plot displaying 42 cases of *DICER1*-mut thyroid cancers including the cases from current study and a review of the literature. Only mutations in the RNase IIIb domain and from malignant tumors were included. Clinicopathologic characteristics include age, sex, histology, N status and M status. The different studies included in the design of this plot are shown. *DICER1* RNase IIIb mutations were categorized according to mutated codon. cPTC, classic papillary thyroid cancer; FTC, follicular thyroid cancer; fvPTC, follicular variant papillary thyroid cancer; PDTC, poorly differentiated thyroid cancer; N, lymph node metastasis status; M, distant metastasis status. **(B)** *DICER1* mutation lollipop plot depicting RNase IIIb mutations from 42 patients with thyroid cancer. **(C)** Expression of selected MAPK output genes present in the HTG Oncology Biomarker Panel in non-neoplastic/hyperplastic vs *DICER1*-mutant cases. **(D)** Expression of genes related to thyroid differentiation in non-neoplastic/hyperplastic vs *DICER1*-mutant cases. Asterisk represent p < 0.05.

In thyroid cancer, genetic alterations activating the MAPK pathway are highly prevalent and mutually exclusive to each other, indicating that harboring more than one of these mutations confers no clonal advantage (17, 29, 35). In this study, all cases with *DICER1* RNase IIIb mutations lacked genetic alterations activating the MAPK pathway. A literature review of 42 thyroid cancers (including cases from this study) harboring *DICER1* RNase IIIb mutations reinforced the mutually exclusive nature of these alterations in thyroid cancer (**Figure 3A**). The *DICER1* mutations reviewed here - all in the RNase IIIb domain - are asymmetrically distributed between codons E1705, D1709, D1810, and E1813. E1813 is the most common alteration (20/42; 48%) overall and in each thyroid cancer type (PTC, FTC, PDTC) (**Figures 3A, B**). Even in more advanced forms of the disease such as PDTC, there was no overlap between *DICER1* hotspot mutations and genetic alterations activating the MAPK pathway (18, 27). This supports the hypothesis that these alterations are associated with MAPK signaling activation. Indeed, mRNA expression of established MAPK output markers *HMGA2*, *PPAT, EGR1* and *CCND1* (from TCGA ERK-score (17)) is increased in *DICER1*-mut tumors when compared to non-neoplastic/hyperplastic thyroids (**Figure 3C**). Although it is not clear how *DICER1* hotspot mutations activate MAPK, we found several positive regulators of the pathway to be upregulated in *DICER1*-mut tumors: *NRG1*, RTKs (*FGFR3*, *ERBB2/3*), *BRAF* and *RAF1.* We also found decreased expression of *TPO*, *TFF3*, *LRP2* and increased expression of *FN1*, genes positively and negatively correlated with differentiation, respectively (**Figure 3D**). Although patients with *DICER1* hotspot mutations have well differentiated thyroid tumors of follicular cell origin and are at low risk for lymph node metastasis, a fraction of cases are associated with high-risk PTC or PDTC (16). Our literature review of *DICER*-mutant cases shows that the one case of PTC with N1b disease harbored a *TP53* truncating mutation (p.E343Afs*3) (**Figure 3A**). Moreover, five out of 12 (41%) *DICER1*-driven PDTC also had a *TP53* mutation (4/12, 33%) or a *TERT* promoter mutation (1/12, 8%), alterations associated with progression of thyroid cancer (**Figure 3A**).

### *DICER1*-mutation impact the expression of genes related to cell-cycle

**Figure 4:**
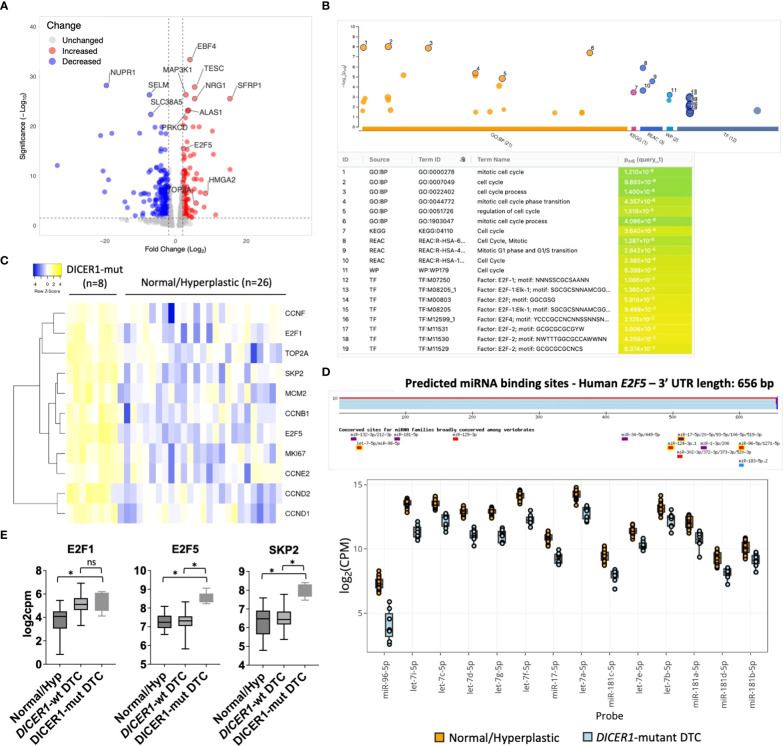
*DICER1* RNase IIIb mutations positively regulate mitotic cell-cycle genes. **(A)**.Volcano plot highlighting differentially expressed mRNAs between *DICER1*-mut differentiated thyroid cancer (DTC) (n=8) vs. non-neoplastic/benign hyperplastic group (n=26). The blue dots denote downregulated gene expression and the red dots denote upregulated gene expression. Grey dots denote gene expression without significant differences (FC>2 and FDR≤.05). **(B)** Functional enrichment analysis using g:Profiler version e106_eg53_p16_65fcd97. Analysis of 247 upregulated genes reveals enrichment for genes related to mitotic cell-cycle in different databases (GO, KEGG, Reactome, Wikipathways) and over-representation of E2F transcription factor binding sites according to TRANSFAC database. **(C)** Heatmap displaying increased expression of *E2F1* and *E2F5* transcription factors and selected genes related to cell-cycle in *DICER1*-mutant tumors vs. non-neoplastic/benign hyperplastic tissues. Yellow: upregulated genes; Blue: downregulated genes. **(D)** Predicted miRNA binding sites in the 3’ untranslated region (UTR) of *E2F5* using TargetscanHuman 8.0 (Top) and downregulation of miRNAs predicted to target *E2F5* 3’ UTR in *DICER1*-mut vs non-neoplastic/hyperplastic tissues (bottom). **(E)** Increased expression of *E2F1*, *E2F5* and *SKP2* mRNA in *DICER1*-mut DTC vs. *DICER1*-wt DTC and non-neoplastic/hyperplastic tissues. Asterisk represent p < 0.05; ns: not significant.

To understand the molecular mechanisms (gene networks and signaling pathways) underlying tumorigenesis of follicular cells harboring *DICER1* mutations, we analyzed the mRNA expression profiles of the same pediatric thyroid tumors using the HTG Edgeseq OBP panel, which evaluates the mRNA expression of 2,559 genes related to cancer (**Supplemental Table 2**). We performed a differential gene expression analysis between eight *DICER*1-mut tumors and 26 non-neoplastic/hyperplastic thyroids and observed 569 differentially expressed genes (DEG) between the two groups (247 up and 322 down, FDR<0.05) (**Figure 4A**). To evaluate the pathways enriched among the DEGs from the attained dataset, we used g:Profiler web-based tool (v11.5) (26). Functional enrichment analysis of these 247 upregulated genes exposed a significant enrichment for cell-cycle genes in four different databases: Gene Ontology (GO), Reactome, KEGG and WikiPathways (**Figure 4B**). Among the upregulated genes in *DICER1*-mutant compared to non-neoplastic and hyperplastic lesions (FC≥2, and FDR≤.05), were genes related to cell-cycle, such as the transcription factors *E2F1* and *E2F5*, different cyclins (*CCNB1*, *CCND1*, *CCND2*, *CCNE2*, *CCNF*), *SKP2*, *TOP2A*, *MCM2*, and the established clinical marker of cell proliferation, *MKI67* (**Figure 4C**). E2F transcription factor binding sites were also found to be significantly overrepresented in the collection of upregulated genes based on TRANSFAC analysis, pointing to these transcription factors as important hubs in the cell-cycle regulation of *DICER1*-mut thyroid tumors (**Figure 4B**). While *E2F1* was upregulated in all tumors (*DICER1*-wt and *DICER1*-mut), *E2F5* was specifically upregulated in *DICER1*-mut tumors. Remarkably, we found that let-7, mir-17-5p, mir-96-5p and mir-181-5p, all of which were found to be downregulated in *DICER1*-mut tumors, have predicted binding sites that are conserved among vertebrates in the 3’ UTR of *E2F5* (**Figures 4D, E**). Indeed, the regulation of *E2F5* mRNA expression by some of these miRNAs has already been previously validated in other cancer types (36–38). The differential expression of selected mRNAs associated with cell-cycle (*E2F5*, *SKP2*), MAPK signaling output (*HMGA2*, *CCND1*) and thyroid differentiation (*TPO*) was further validated by quantitative PCR in four *DICER1*-mut DTC and matched normals – cases ID# 5, 6, 7 and 8 from **Table 1** (**Supplemental Figure 4**).

## Discussion

DICER1 is a highly conserved RNase III endoribonuclease which plays a critical role in the biogenesis of miRNAs. Several studies have reported biallelic *DICER1* variants in syndrome-related tumors as well as *DICER1*-driven sporadic tumors (20, 22, 39), however the mechanisms by which the mutation affects miRNA and mRNA transcriptomes remain to be fully elucidated. Pediatric thyroid tumors are a particularly interesting model to dissect the effects of *DICER1* mutations on miRNA and mRNA changes. These tumors have a relative quiet genome, with low mutational burden (40). Moreover, *DICER1*-mut DTC typically lack other tumor-initiating driver mutations, suggesting that *DICER1* mutations are the sole drivers of these tumors. The lack of additional genomic changes in these tumors affords a clear panorama of the miRNA and mRNA changes triggered by *DICER1* mutations. In fact, our analysis uncovered a clinically relevant miRNA signature in *DICER1*-mut malignant nodules that could help guide thyroid nodule diagnosis and management. The downstream changes in gene expression caused by *DICER1* mutations and miRNA dysregulation revealed an important role of the RNase IIIb mutation in the activation of the MAPK pathway, loss of differentiation and positive regulation of cell-cycle.

*DICER1* RNase IIIb mutations are found in both benign and malignant follicular derived thyroid tumors, predominantly in follicular-patterned lesions. Even when malignant, *DICER1*-mut tumors are associated with less invasive disease and generally have an excellent prognosis, clinically resembling *RAS*-mutant thyroid tumors. However, *DICER1*-mut DTC can progress to PDTC, similar to *RAS*-mutant tumors (16, 27). Therefore, it is critical to add molecular layers of information to improve the risk assessment of these thyroid neoplasms. The set of 3p miRNAs found to be specifically upregulated in *DICER1*-driven malignant tumors may be effective markers to discriminate the malignant status of nodules harboring the RNase IIIb mutation.

As we expand our multi-institutional collaborations within the CATC, it will be critical to evaluate a greater number of benign and malignant tumors with RNase IIIb mutations to validate these findings. The detection of 3p miRNAs associated with *DICER1* mutations provides a promising path to enhance the diagnostic risk of malignancy in thyroid nodules. It is worth noting that commercially available thyroid-specific miRNA panels should be used with caution in pediatric thyroid cancers where *DICER1* mutations are more frequent (41). Some miRNAs included in these panels, such as mir-146b-5p and mir-31-5p, are consistently increased in malignant thyroid disease but found to be markedly reduced in *DICER1*-mut DTCs based on data from this study as well as the Thyroid Cancer TCGA (17). Relying on these panels to assess the risk of malignancy could increase the possibility of a benign call for a *DICER1*-mut malignant nodule.

*DICER1* mRNA itself has a long 3’ UTR (~4,000 nucleotides) which allows multiple miRNAs to bind and repress its expression. Some miRNAs that have been reported to target the *DICER1* 3’ UTR include let-7 (42), miR-9 (43), and miR-146b-5p (44), all of which are 5p miRNAs downregulated in *DICER1*-mut tumors. These findings point to a negative feedback mechanism in the regulation of *DICER1* expression that could explain the increase in *DICER1*, and consequently, the increase in 3p miRNAs observed in *DICER1*-mut cases.

In thyroid cancer, *DICER1* hotspot mutations are mutually exclusive with MAPK mutations, suggesting their ability to activate this pathway. Indeed, we found increased expression of positive modulators of the pathway as well as MAPK signaling output markers. Because the mRNA expression analysis utilized in this study was limited to the ~2,500 genes included in the HTG OBP panel, we were unable to assess the ERK score (52-gene list signature) and the Thyroid Differentiation Score (TDS, 16-gene list signature) generated by the Thyroid Cancer TCGA to evaluate MAPK signaling output and thyroid differentiation, respectively (17). However, two previous reports of integrated mutational and gene expression analysis with two *DICER1*-mut cases reported increased MAPK output and decreased TDS for *DICER1*-mut tumors (17, 45). In both studies, MAPK output and TDS scores were intermediate between non-neoplastic and *BRAF*/fusion-positive tumors, similar to the RAS-like subgroup of tumors from the thyroid cancer TCGA (well-differentiated tumors with favorable prognosis), strongly suggesting that RNase IIIb mutations have the ability to activate the pathway. Interestingly, this appears to be exclusive to thyroid cancers as in other tumor types such as endometrial cancers, pleuropulmonary blastomas, sarcomas, Sertoli-Leydig cell tumor, colorectal cancer, and cutaneous melanoma, *DICER1* hotspot mutations frequently co-occur with MAPK alterations and/or other driver alterations (10, 46).

The enrichment of cell-cycle genes found in *DICER1*-mutant tumors agrees with previous reports demonstrating that DICER1 RNase IIIb mutations seem to confer a proliferative advantage to affected cells (47, 48). Although sustaining proliferative signaling is a hallmark of cancer, the unique mechanism among these thyroid tumors indicates that E2F transcription factors, specially *E2F5*, may have an important role in this process. Moreover, the increased expression of the S-phase kinase protein 2 *SKP2* (also specific to *DICER1*-mut tumors), a major ubiquitin ligase that controls abundance of cell-cycle regulatory proteins at the G_1_–S transition may be an important target for therapies of these tumors (49), particularly when progression to high-grade PTC or PDTC occurs. This study highlights a previously unreported mechanism by which *DICER1* mutations may induce transformation, and suggest novel therapeutic approaches for treating these rare malignancies.

The rarity of tumors bearing *DICER1* variants represents a significant challenge to studying the role of *DICER1* mutations in oncogenesis. Most studies report no more than two or three cases harboring the RNase IIIb mutation, making it difficult to correlate these alterations with clinical features and molecular changes. The CATC is building a collaborative research community dedicated to studying thyroid cancers and other thyroid-related conditions, including the establishment of a biorepository, in pediatric patients. Through these initial efforts and networks within the CATC, we were able to collect eight cases. Future studies will seek to leverage the growing network of the CATC and to utilize the signature reported here to identify additional *DICER1*-mutant tumors (which are enriched in the pediatric population) to further elucidate the mechanisms of *DICER1*-induced transformation.

To our knowledge, there are no commercially available cancer cell lines with spontaneous hotspot mutation in the RNase IIIb domain of *DICER1* (50). In addition, different mouse models and human cell lines have been established to study *DICER1* effects in cancer by simply knocking out the gene. The generation of proper models inactivating the RNase IIIb domain of DICER1 while preserving function of the RNase IIIa will be important for proper mechanistic studies of *DICER1-*related tumors.

In conclusion, our integrated analysis of mutational data, miRNAs, and mRNA profiling suggests that DTC with *DICER1* RNase IIIb hotspot mutation generate an unbalanced expression of 5p:3p miRNAs that could be valuable for diagnostic and prognostic purposes. These tumors are associated with an increase in MAPK output and decreased differentiation, resembling the RAS-like tumors subgroup from the adult thyroid cancer TCGA, which is correspondingly reflected in their clinically indolent behavior. Mechanistic studies will be necessary to further understand the pathways and biological networks associated with the *DICER1*-driven changes in miRNAs, especially those changes in 3p miRNAs, which seem to be relevant in malignant progression.

## Data availability statement

The data presented in the study are deposited in the NCBI repository, Bioproject ID: PRJNA934932 (https://www.ncbi.nlm.nih.gov/bioproject/?term=PRJNA934932).

## Ethics statement

The study was approved by Children’s Hospital of Philadelphia Institutional Review Board (IRB# 20-018240). Written informed consent to participate in this study was provided by the participants’ legal guardian/next of kin.

## Author contributions

JR-F, AF, JW, and AB designed the research. JR-F, AF, JW, and AB conducted the research. JR-F and AF wrote the article and JR-F had primary responsibility for final content. Financial support: AF, AB, JW, and DS; Administrative support: AF, AI, AB, and MS; Provision of study materials or patients: AB, JW, AI, TP, and JB. All authors contributed to the article and approved the submitted version.
