# Supplementary figures and images for: DICER1 RNase IIIb domain mutations trigger widespread miRNA dysregulation and MAPK activation in pediatric thyroid cancer
Source: Front Endocrinol (Lausanne). 2023 Feb 21;14:1083382. doi: 10.3389/fendo.2023.1083382 (PMC9990750; doi:10.3389/fendo.2023.1083382)

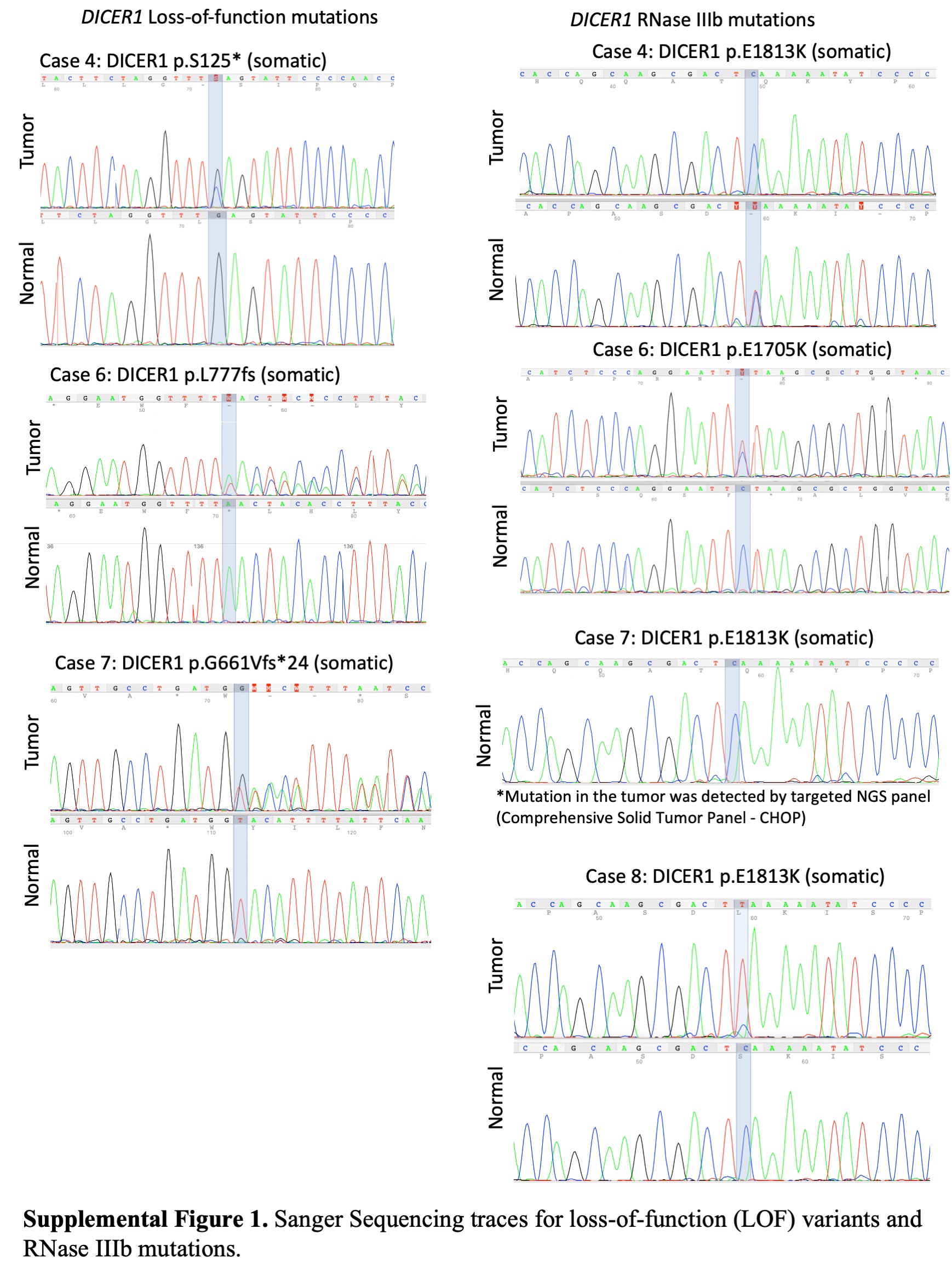

Supplement: Supplementary file 1 [file Image_1.jpeg]

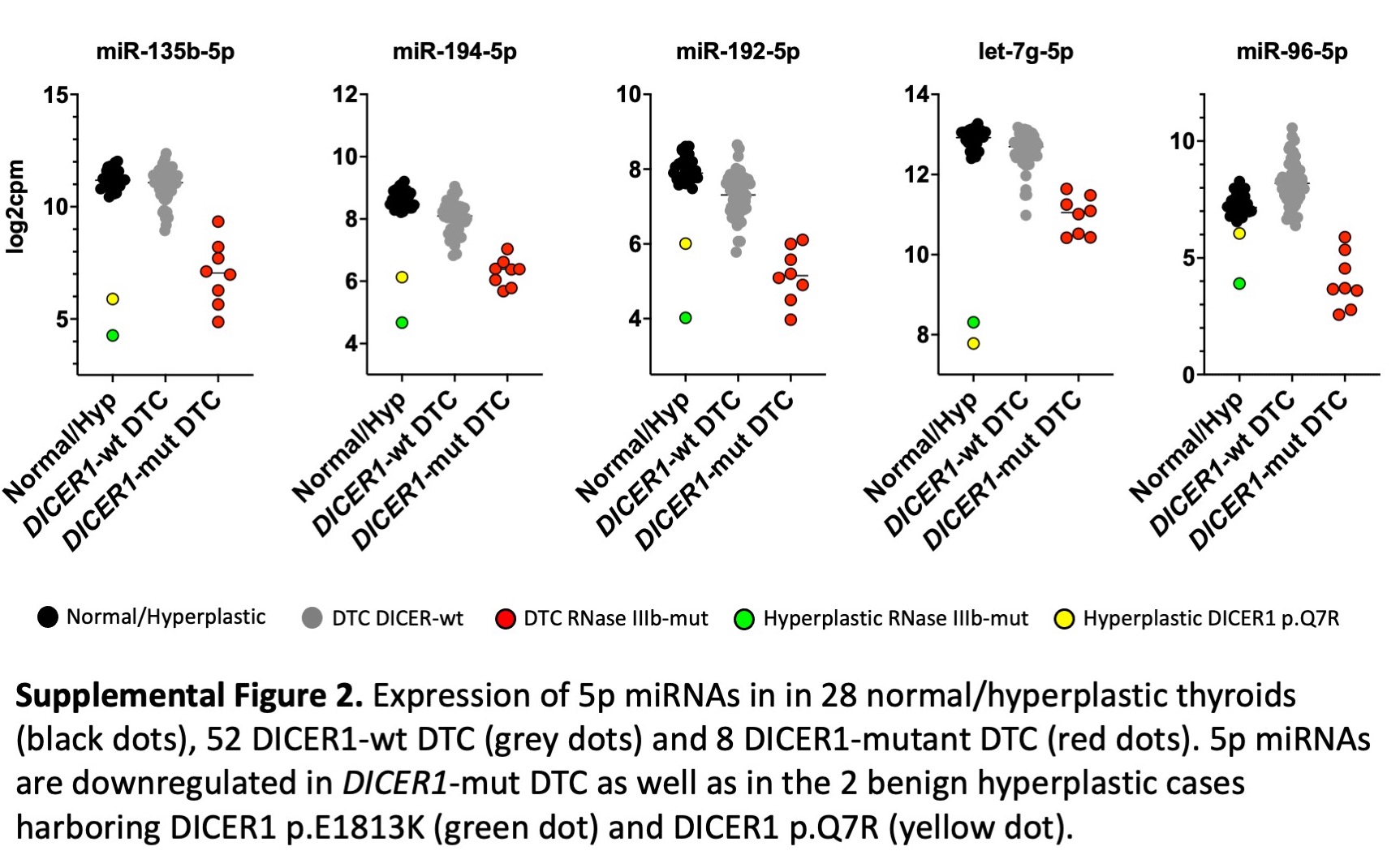

Supplement: Supplementary file 2 [file Image_2.jpeg]

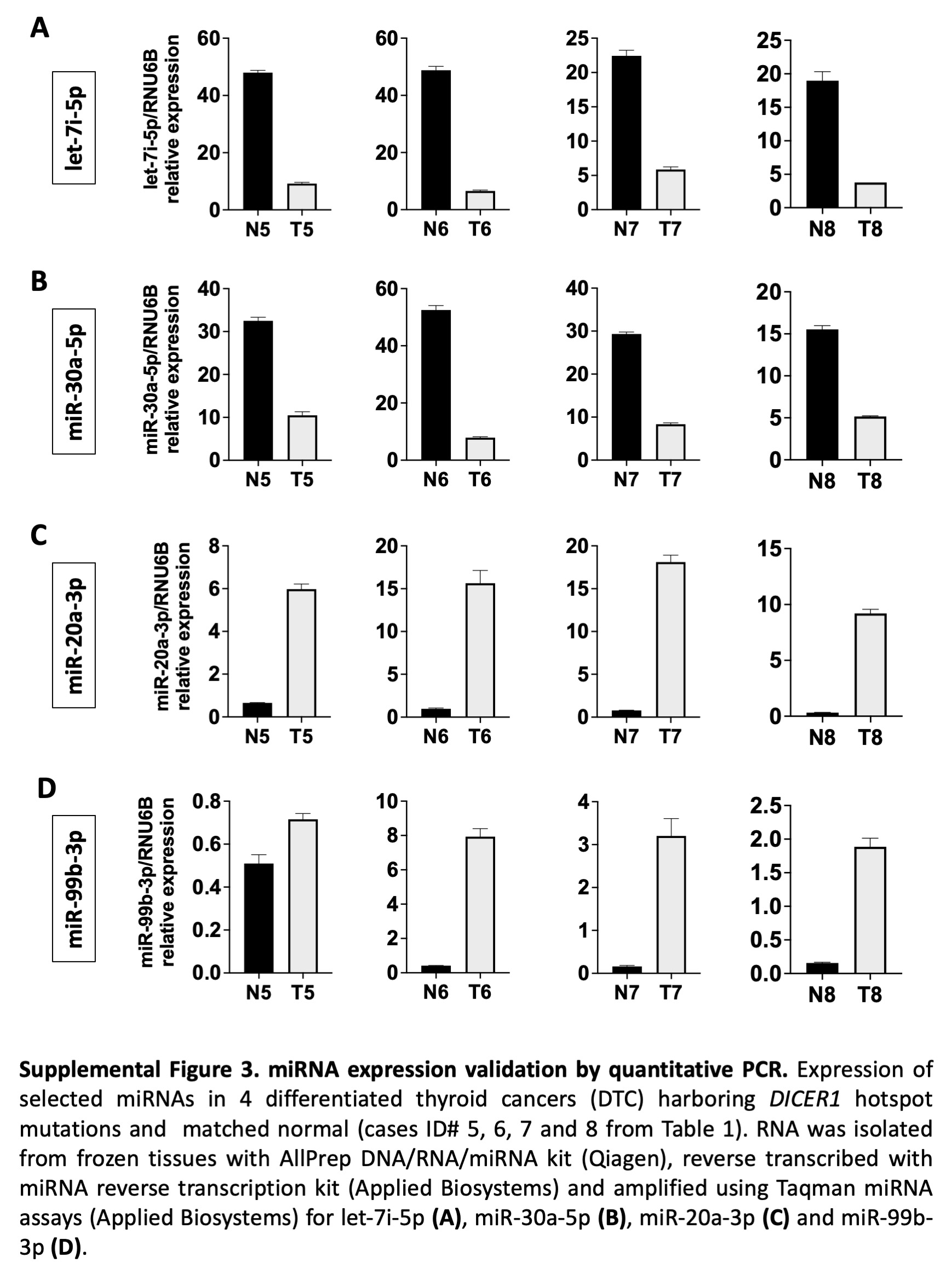

Supplement: Supplementary file 3 [file Image_3.jpeg]

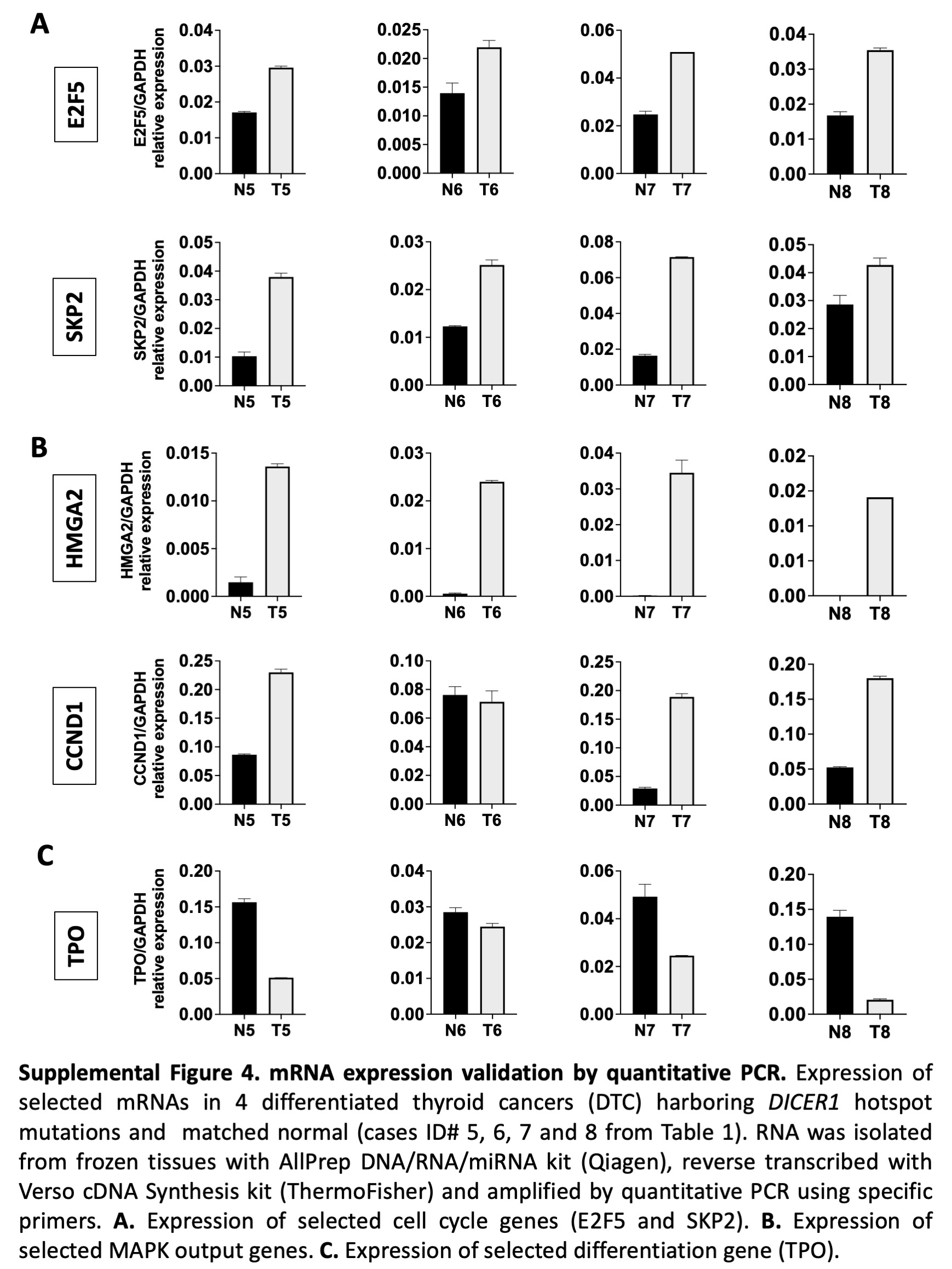

Supplement: Supplementary file 4 [file Image_4.jpeg]
